# Psychotropic Medications for Non-Psychiatric Conditions: A Narrative Review

**DOI:** 10.3390/healthcare13172122

**Published:** 2025-08-26

**Authors:** Melanie T. Gentry, Jonathan G. Leung, Laura Suarez, J. Michael Bostwick

**Affiliations:** 1 Department of Psychiatry and Psychology, Mayo Clinic, 200 First Street SW, Rochester, MN 55905, USA; suarez.laura@mayo.edu (L.S.); bostwick.john@mayo.edu (J.M.B.); 2 Department of Pharmacy, Mayo Clinic, Rochester, MN 55905, USA; leung.jonathan@mayo.edu

**Keywords:** polypharmacy, psychopharmacology, drug interactions

## Abstract

Since the discovery of the first psychoactive medications, their psychiatric and medical uses have overlapped. Designating a medication psychiatric or psychotropic is thus arbitrary, based on its most common usage, but labeling it so obscures the full range of its pharmacologic activity and clinical utility. Psychotropic medications (PMs) are frequently used to treat medical conditions or symptoms in the areas of neurology (e.g., migraine, seizure disorder), dermatology (e.g., itching), gastroenterology, and chronic pain, among many others. It is important for both primary care and specialty physicians to be aware of the potential non-psychiatric indications for the use of common PMs such as antidepressants, antipsychotics, and anxiolytics. This review examines the potential benefits and risks of PMs when used for non-psychiatric indications, highlighting how their pharmacologic effects on neurotransmitters contribute to both therapeutic outcomes and adverse effects. It also provides guiding principles for prescribers, including the importance of adjusting doses based on the specific indication, monitoring for harmful side effects, considering age-related factors, and addressing the risks associated with polypharmacy.

## 1. Introduction

In 1754, Horace Walpole, the English polymath, became intrigued by a Persian folktale in which three princes of Serendip traveled the world, “making discoveries, by accident and sagacity, of things they were not in quest of.” Inspired by this tale, Walpole coined the term serendipity to describe the fortunate occurrence of making unexpected discoveries [1]. He could well have been anticipating, two centuries in advance, the foundations of psychotropic pharmacotherapy. In the 1950s, serendipity played a pivotal role when chlorpromazine, originally developed as a surgical anesthetic, was found to significantly reduce agitation, paranoia, and hallucinations, thereby revolutionizing psychiatric care [2]. Similarly, the first antidepressant was identified when patients treated with the antitubercular agent iproniazid experienced unanticipated improvements in mood, appetite, and sleep [3]. Though developed for tuberculosis, iproniazid was soon used off-label to treat depression. In the 2000s, Vietnam veterans prescribed the alpha-1 blocker prazosin for benign prostatic hypertrophy reported a marked reduction in the frequency and intensity of trauma-related nightmares [4]. In each of these instances, psychiatric breakthroughs emerged through the serendipitous discovery of unexpected therapeutic effects in medications not originally intended for psychiatric use.

Conversely, medications originally approved for psychiatric indications have found increasing use in other medical specialties. Between 2014 and 2015, nearly 380 million psychotropic medications (PMs) were prescribed in the United States [5]. Antidepressants rank among the top three most prescribed therapeutic drug classes, with 12.7% of individuals aged 12 and older reporting antidepressant use in the past month [6]. While the high prevalence of mental health disorders such as major depressive and generalized anxiety disorders partially explains this volume, it does not fully account for it, suggesting a substantial degree of off-label use.

Pharmaceutical companies have come under increased scrutiny for promoting such serendipitously discovered uses without obtaining Federal Drug Administration (FDA) approval for the new indications. In 2012, for example, GlaxoSmithKline was fined $3 billion by the U.S. government, in part for the off-label marketing of several drugs, including paroxetine [7]. While patients and clinicians may find off-label use of PMs beneficial, cautious and informed prescribing is essential, particularly considering the potential side effects and risks of polypharmacy (see Table 1).

PMs have the potential for both pharmacodynamic and pharmacokinetic interactions with other medications. They have complex mechanisms of action and may compete for, inhibit, and induce the metabolism of other simultaneously prescribed medications. This requires a careful understanding of PMs pharmacological properties. A thorough understanding of the mechanisms of action of key neurotransmitters, like dopamine, norepinephrine, and serotonin, along with the increasing prominence of GABA and glutamate, is essential for assessing both therapeutic benefits and adverse effects when these agents are used beyond traditional psychiatric indications. Additionally, many psychiatric medications, including tricyclic antidepressants (TCAs) and antipsychotics, act on histamine-1, muscarinic, and alpha-adrenergic receptors, contributing to their broad clinical effects while also increasing the risk of adverse side effects. Table 2 illustrates these receptor interactions and their associated clinical implications.

Furthermore, many non-PMs possess secondary mechanisms that influence monoaminergic pathways, potentially leading to drug interactions or adverse events when used in combination with PMs. For example, cyclobenzaprine and fentanyl have serotonergic properties and may increase the risk of serotonin syndrome when co-administered with serotonergic antidepressants. Several antiemetics, including prochlorperazine, in the same class as first-generation antipsychotics like chlorpromazine, exhibit dopamine-blocking effects and may amplify the risk of dopamine antagonism-related adverse effects when combined with antipsychotic medications (e.g., dystonia, akathisia). When prescribing combinations of PMs and non-PMs, clinicians should provide thorough patient counseling and document potential risks, particularly serious adverse outcomes such as serotonin syndrome, neuroleptic malignant syndrome, and tardive dyskinesia. This review examines four medical specialties to highlight how PMs, when used for non-psychiatric indications, can result in therapeutic benefit and/or clinically significant side effects.

### Psychotropic Medication Use by Specialty

There are multiple and complex factors that relate to the use of PMs across different specialties. Many, though not all, of the medical diagnoses discussed in this review have high rates of comorbid psychiatric disorders, suggesting a complex interplay. This may reflect bidirectional relationships, wherein the stress of chronic illness contributes to the development of depression or anxiety symptoms, or where shared physiological pathways and risk factors predispose individuals to both psychiatric and medical conditions.

Importantly, PMs may also confer benefit in the absence of any psychiatric comorbidity, suggesting broader therapeutic potential. As each specialty is examined, the proposed mechanisms of action by which PMs may influence medical conditions will be explored. In many instances, the underlying mechanisms have yet to be fully elucidated. Given the breadth of specialties addressed, this review does not aim to provide a comprehensive or systematic evaluation of the evidence supporting each off-label indication. Rather, it offers a broad overview of the potential risks and benefits, based on current literature, and does not constitute a recommendation for off-label prescribing.

## 2. Methods

This review seeks to answer the following questions: why are PMs so commonly used in non-psychiatric medical specialties? Are they simply addressing co-occurring psychiatric illnesses such as depression and anxiety disorders, or do they exert a direct therapeutic effect on the core symptoms of various medical conditions? What guidance do primary care and specialty clinicians require in prescribing PMs? These broad questions are not well described in the existing literature and require a complex synthesis informed by clinical experience. Due to the multiple diagnoses and specialties included, there were multiple literature searches performed, targeted toward each individual specialty area. The searches were performed using PubMed with search terms such as psychotropic medications, antidepressants, serotonin reuptake inhibitors, cross referenced with migraine headache, vestibular disorders, GI conditions, and pain. A variety of study designs were included for review. Prioritization was given to well-designed systematic reviews, meta-analyses, and clinical practice guidelines for each or the conditions reviewed. The search did not limit the time period, but priority was given to the most recent and relevant research studies. The search included only studies published in English and excluded unpublished studies and conference abstracts.

## 3. Results

### 3.1. Neurology

Both neurology and psychiatry deal with brain pathology and posit neurotransmitter imbalances as explanations for what can be overlapping manifestations of disease. Although both specialties utilize PMs, they often do so for distinct indications, which can lead to confusion among patients and clinicians regarding the purpose of a given prescription. For example, valproic acid may be prescribed for either bipolar disorder or seizure disorders, while TCAs may be used to treat migraine, major depressive disorder, or both. When patients carry both psychiatric and neurologic diagnoses, they may be prescribed multiple PMs for different indications, thereby increasing the risk of drug–drug interactions and polypharmacy [8]. Clinicians must remain vigilant to these risks and carefully assess potential interactions when introducing any additional PM, regardless of the primary indication.

### 3.2. Neurologic Conditions

#### 3.2.1. Headache

The exact pathophysiology of migraine remains poorly understood, though it is thought to involve multiple mechanisms, such as heightened excitatory signaling, impaired GABAergic inhibition, cortical spreading depression, neuronal sensitization, and elevated calcitonin gene-related peptide (CGRP) levels [9]. Preventive treatment targets these pathways using various agents, including antidepressants, antiepileptics, antihistamines, beta-blockers, and calcium channel blockers [10].

Antidepressants have demonstrated efficacy in headache prevention through modulation of norepinephrine and serotonin reuptake, as well as anticholinergic, histaminergic, and GABAergic effects [11,12]. Amongst PMs, the strongest evidence has been found for TCAs in migraine prevention. A recent meta-analysis showed amitriptyline to be superior to fluoxetine and comparable to antiepileptics like topiramate and valproate in migraine prophylaxis [11]. While SSRIs and SNRIs show indication of benefit vs. placebo in meta-analyses, the evidence is based on lower quality evidence with underpowered trials, often limited by small sample size [13]. Notably, amitriptyline is effective at low doses (10–60 mg), often below those used for depressive disorders [14]. When depression coexists, underdosing may be mistaken for treatment failure, while unrecognized duplication with other antidepressants increases the risk of adverse effects. Divalproex sodium (valproate) is effective and generally well tolerated for both migraine and cluster headache prevention [15]. Its mechanisms include GABA enhancement, ion channel blockade, CGRP downregulation, and inhibition of protein kinase C [10]. Lithium may also help with cluster headaches, though its narrow therapeutic index, requirement for monitoring, and frequent side effects limit its utility [16].

Antipsychotic medications have antiemetic properties and can help mitigate nausea and pain in acute migraine episodes. Studies of intravenous chlorpromazine and haloperidol have shown them to be more effective for pain relief than placebo, but with a high rate of adverse effects including orthostatic hypotension, sedation, and akathisia [17,18].

#### 3.2.2. Parkinson’s Disease

In addition to its hallmark motor symptoms, Parkinson’s disease (PD) is associated with multiple non-motor manifestations, including depressive symptoms, psychosis, impulse control disorders, and insomnia. PMs are commonly used to address these symptoms. Notably, selective MAO-B inhibitors such as selegiline and rasagiline not only address psychiatric symptoms but also improve core motor features of PD by increasing synaptic dopamine and inhibiting its degradation [19]. Although selegiline was discovered in the 1960s, it did not receive FDA approval for PD until 1989, and its use for depression came much later. At standard oral doses for PD (≤10 mg), selegiline has low bioavailability and is generally ineffective for treating depressive disorders unless doses are escalated [20]. However, its transdermal formulation, which offers higher systemic exposure, was approved in 2006 for major depressive disorder. Rasagiline, a newer and more expensive MAO-B inhibitor, has been promoted for potential, but unproven, neuroprotective effects in PD [19]. It carries potential contraindications when used in combination with serotonergic agents such as St. John’s Wort, cyclobenzaprine, certain opioids, and other MAOIs, due to the risk of serotonin syndrome [21]. Nevertheless, two clinical trials reported the safe use of low-dose antidepressants alongside rasagiline without significant adverse interactions [22].

#### 3.2.3. Vestibular Disorders

There is emerging evidence that selective serotonin reuptake inhibitors (SSRIs) may aid in the management of certain vestibular disorders by modulating serotonergic pathways involved in the brain’s processing of balance, motion, and spatial orientation. SSRIs may help reduce vestibular hypersensitivity, anxiety-related dizziness, and functional symptoms that persist following an initial vestibular insult. Conditions in which SSRIs and serotonin-norepinephrine reuptake inhibitors (SNRIs) may be beneficial include Persistent Postural Perceptual Dizziness (PPPD), vestibular migraine, and Mal de Débarquement syndrome [23,24]. However, robust evidence from randomized controlled trials remains limited.

### 3.3. Gastroenterology

#### 3.3.1. Functional Gastrointestinal Disorders (FGIDs)

In recent decades, gastroenterologists have increasingly recognized the brain–gut interaction in diagnosing and managing functional gastrointestinal disorders (FGIDs). While serotonin is well known for its role in mood, sleep, and appetite, the majority is produced in the gastrointestinal (GI) tract, where it regulates motility, secretion, inflammation, and sensation via the enteric nervous system (ENS) [25]. The bidirectional communication between the ENS and the central nervous system (CNS) occurs through hormonal, neural, and immune pathways, allowing PMs to exert both beneficial and adverse GI effects [26].

Historically, the link between psychological factors and GI symptoms has been well documented, with older terms like nervous dyspepsia reflecting debates over whether such conditions were primarily psychiatric or GI [27]. FGIDs are now well recognized as distinct syndromes, though PMs remain integral to their management [28]. Various PM classes are used based on symptom profile and psychiatric comorbidity. TCAs are particularly effective for pain-predominant FGIDs (e.g., centrally mediated abdominal pain syndrome, functional chest pain, functional dyspepsia, and anorectal pain), and their anticholinergic effects can reduce diarrhea in irritable bowel syndrome (IBS) diarrhea-predominant [29]. SSRIs may be preferred for IBS with constipation, non-painful FGIDs, and patients with coexisting anxiety, depression, or somatic symptom burden. Buspirone, a 5-HT1A receptor partial agonist, improves gastric accommodation by enhancing vagal tone and modulating enteric serotonergic signaling. It promotes fundus relaxation, helping to reduce symptoms like early satiety, bloating, and postprandial fullness in conditions such as functional dyspepsia and gastroparesis [30].

Meta-analyses support the effectiveness of PMs in treating IBS, though they are associated with a higher incidence of adverse effects compared to placebo [31]. Among patients with FGIDs, particularly those with comorbid psychiatric conditions, negative perceptions of PMs are common [32]. As such, while PMs may offer meaningful therapeutic benefits in FGIDs, mental health stigma can pose a barrier to their acceptance and adherence for some individuals.

#### 3.3.2. Antiemetics

Some PMs are frequently used for their antiemetic properties. Mirtazapine enhances norepinephrine and serotonin release through central α_2_-adrenergic antagonism and blocks 5-HT2A, 5-HT2C, and 5-HT3 receptors while preserving 5-HT1A activity. These mechanisms contribute to its antidepressant, anxiolytic, and antiemetic effects. Its potent H_1_ receptor antagonism also explains its sedative and appetite-stimulating properties. Olanzapine provides antiemetic benefit by antagonizing dopamine D_2_, serotonin 5-HT2 and 5-HT3, histamine H1, and muscarinic receptors, thereby targeting multiple pathways involved in nausea and vomiting [33,34].

### 3.4. Dermatologic

Dermatologic and psychiatric disorders frequently overlap, with delusional parasitosis representing one of the most striking examples. These conditions are broadly categorized as primary psychodermatologic disorders, such as delusional parasitosis, trichotillomania, and pathologic skin picking, or secondary psychodermatologic disorders, where psychiatric symptoms arise in response to a skin condition, such as acne vulgaris leading to social anxiety [35]. The psychodermatologic spectrum also includes more subtle and complex presentations, such as idiopathic urticaria and burning mouth syndrome [36]. A 2019 review of 25 studies found that over 30% of patients with chronic urticaria had a co-occurring psychiatric disorder [37]. Stress and anxiety can worsen common skin conditions including eczema, psoriasis, rosacea, and acne; while scarring or disfigurement can negatively impact self-esteem, body image, and psychosocial functioning, contributing to depression and anxiety [38].

PMs are commonly used and may offer substantial benefit in managing psychodermatologic conditions due to their multiple mechanisms of action. Antihistamines (e.g., hydroxyzine, diphenhydramine) and TCAs have antipruritic and anxiolytic effects. Additionally, there is growing recognition of the anti-inflammatory properties of certain antidepressants [39]. Doxepin is often used for idiopathic itching and urticaria, amitriptyline for burning or stinging pain, and topical ketamine/amitriptyline formulations for neuropathic pruritus [39,40]. Many patients, especially those with delusional parasitosis, may resist psychiatric referral, leaving dermatologists to initiate PMs. Traditionally, pimozide has been used, though newer second-generation antipsychotics like olanzapine and risperidone also show promise [41]. However, the evidence base for PMs in dermatologic conditions is of low to moderate quality with primarily open label or case series studies. As a result, PMs are most appropriate for refractory cases. Their use should follow careful clinical consideration and, ideally, occur within a multidisciplinary setting. When appropriate, evidence-based behavioral treatments should also be incorporated into the care plan [42,43].

### 3.5. Pain Medicine

#### 3.5.1. Chronic Pain

Chronic pain (CP) is associated with significant disability, reduced quality of life, and high rates of comorbid anxiety and depressive disorders [44]. Amid growing concern over opioid misuse, non-opioid strategies, including PMs, have become increasingly important as primary or adjunctive treatments. CP arises from a range of conditions, neuropathy, chronic low back pain, fibromyalgia, migraine, and FGIDs, with evidence-based pharmacologic strategies varying by diagnosis.

Antidepressants, particularly TCAs and SNRIs, are commonly used for pain relief independent of their antidepressant effects [45]. Their mechanisms include enhancement of descending inhibitory pathways and inhibition of glutamate and substance P release in the spinal cord [46,47]. TCAs may be effective at lower doses than those used for depressive disorders but are limited by side effects such as cardiac toxicity, orthostatic hypotension, dry mouth, constipation, and urinary retention, even at these lower doses [45,48]. This has increased interest in the use of SNRIs and SSRIs. Although SSRIs modulate pain pathways, they have not gained a major role in CP management. A 2016 systematic review reported positive outcomes with fluoxetine in fibromyalgia, migraine, and diabetic neuropathy [49]. Other systematic reviews found that SSRIs had inconsistent findings and were of low methodological quality [49,50]. SSRIs may still be useful in cases with comorbid anxiety or depressive disorders, especially if other treatments fail.

#### 3.5.2. Neuropathic Pain

For neuropathic pain, TCAs, SNRIs, and calcium channel-modulating anticonvulsants (e.g., gabapentin, pregabalin) are first-line therapies [51]. While TCAs are often preferred, growing evidence supports SNRIs, particularly duloxetine, for their similar efficacy and better tolerability [52]. Duloxetine is FDA-approved for diabetic neuropathy, fibromyalgia, osteoarthritis, and chronic musculoskeletal pain [53]. Multimodal therapy, often used to reduce opioid reliance, may include combinations of antidepressants, anticonvulsants, muscle relaxants, and benzodiazepines [54]. However, such polypharmacy increases the risk of sedation, cognitive impairment, and falls, with older adults being particularly susceptible to these adverse effects [55].

## 4. Discussion

Alternative indications of medications based on clinical effects or pharmacologic mechanisms are not new and span all medical specialties. PMs are frequently prescribed for a wide range of indications beyond traditional psychiatric diagnoses. Regardless of the primary indication, all prescribers of PMs must adopt thoughtful and informed prescribing practices, with careful attention to appropriate dosing, therapeutic duplication, and the potential for adverse effects and polypharmacy (see Table 1).

### 4.1. Medication Classification and Labeling

Despite common tendencies to assign medications to specific specialties, such classifications are often arbitrary and misleading. The fact that a pharmaceutical company has sought to market a PM for which is has received FDA approval by no means limits potential judicious use of that drug for so-called off-label use. For example, “antidepressants” are used to treat not only depressive disorders, but also anxiety disorders, post-traumatic stress disorder, and obsessive–compulsive disorder. In recognition of this issue, the European College of Neuropsychopharmacology proposed a pharmacologically based nomenclature, referring to drugs by their receptor targets (e.g., olanzapine as a D2/5-HT2 antagonist) rather than clinical labels [56]. This mirrors terminology used for other drugs, such as describing propranolol as a beta-blocker rather than solely an antihypertensive. Despite support from major publishers, this system has yet to gain widespread adoption, in part due to the FDA’s continued use of traditional classifications. Clinicians should be aware of “hidden” psychiatric medications, drugs with psychiatric effects that may not be labeled or marketed as such, and evaluate their full pharmacologic profiles, not just their most familiar indications.

The term off-label refers to prescribing medications for indications that have not been approved by the FDA (or corresponding regulatory body outside the US) as well as using dosage amounts or forms not specified in labeling information. The FDA provides crucial oversight to ensure efficacy and safety for patients. However, an approval process that relies on profit-driven corporations with the financial resources to develop, research, and market new medications for specific indications only presents a number of biases and limitations in the labeling process. Providing care to patients with strict adherence to FDA-approved indications would be untenable, leaving significant gaps in treating complex patients. Opinions differ amongst clinicians about the importance of FDA labeling in providing optimal medical care to patients [57]. Off-label prescribing is particularly common in rare diseases, psychiatric conditions, and less studied populations (geriatric and pediatric) [58]. While off-label use may have benefits to these patients, wide-spread off-label use may reduce the pressure to pursue rigorous clinical trials once their use in clinical practice becomes well-accepted. Additionally, post-market experience with off label prescribing has often led to the later pursuit of expanded FDA indications. Given that the evidence base for off-label prescribing of PMs may be limited or even speculative, these limitations should be made clear to patients in making informed decisions and in setting realistic expectations.

### 4.2. Safety Considerations

Depending on a particular PM’s profile, it can cause side effects related to their anticholinergic, serotonergic, and dopaminergic properties, as well as potential for corrected QT interval (QTc) prolongation. Anticholinergic effects, such as dry mouth, constipation, urinary retention, confusion, and sedation, are common with agents like paroxetine, TCAs, hydroxyzine, and olanzapine. These effects may be compounded by non-psychiatric medications with anticholinergic activity (e.g., diphenhydramine, oxybutynin, orphenadrine). A high anticholinergic burden is associated with cognitive decline, functional impairment, and increased risk of dementia or delirium, a particular concern in older patients and those with less cognitive reserve [59,60].

Serotonin syndrome is another serious concern. In 2016, the FDA issued a safety warning for opioids when used in combination with other serotonergic agents [61]. Characterized by autonomic instability, neuromuscular hyperactivity, and mental status changes, serotonin syndrome can range from mild to life-threatening. It is most often seen when multiple serotonergic drugs are used together, including antidepressants, fentanyl, cyclobenzaprine, tramadol, and dextromethorphan [62]. Clinicians must remain vigilant when combining these agents. Because symptoms may be subtle and easily overlooked, careful prescribing and close monitoring are essential [63].

The multifaceted mechanisms of antipsychotics explain their use, both on- and off-label, for conditions like nausea, hiccups, and delirium. These agents antagonize not only dopamine D_2_ receptors, but also histamine, muscarinic, and alpha-1 receptors, contributing to sedation, anticholinergic effects, and orthostatic hypotension. Extrapyramidal symptoms such as dystonias, akathisia, and parkinsonism may occur acutely, while tardive dyskinesia, a potentially irreversible condition, can develop with long-term use. Risk is heightened when combined with dopamine-blocking agents like metoclopramide or prochlorperazine. Older adults are particularly vulnerable to these side effects.

To varying degrees, PMs may influence the QTc, which may not be clinically relevant with monotherapy application of PMs. However, with polypharmacy, advanced age, or in the setting of cardiovascular comorbidities, the assessment of potential safety risk (e.g., Torsade de Pointe) needs to be considered. In addition to possible pharmacodynamic interactions with other medications, pharmacokinetic interactions of PMs also need to be evaluated. Many PMs are metabolized by cytochrome P450 (CYP) 2D6 and CYP3A4 or may inhibit certain isozymes, thus setting up the potential for unexpectedly high or low blood levels depending on an individual’s particular cytochrome P450 receptor profile or drug–drug interactions when one drug affects the metabolism of another.

### 4.3. Patient Communication and Shared Decision Making

Although PMs are often used to treat non-psychiatric conditions and for indications not approved by the FDA, prescribers must carefully weigh the risks, benefits, and alternatives before proceeding. They should also thoughtfully consider how much information to share with the patient—particularly whether to disclose that the medication is being prescribed off-label—as part of the shared decision-making process. Shared decision-making is a bidirectional process with clinicians and patients working collaboratively in creating a treatment plan. Clinicians must provide patients with adequate clinical information, tailored to their individual needs, values and preferences in order to prioritize patient autonomy. Therefore, there is no single answer as to what information to provide each patient. However, there are core features of shared decision-making that can be used to guide implementation of this process in clinical practice. With regard to PMs for non-psychiatric conditions, it is important to disclose the common psychiatric indications of these medications, particularly as there may be significant stigma associated with those psychiatric conditions about which patients should be warned. Prescribers may want to further clarify differences in common dosage forms between different indications. Special consideration should be given to the potential adverse effects of PMs in higher risk populations, such as older adults or those with multiple, chronic medical conditions. Increased attention should be given to the risks associated with polypharmacy in these individuals [64,65].

## 5. Limitations

This narrative review has several limitations. It is not intended to be comprehensive, given the vast range of possible alternate uses of PM, but it does illustrate a paradigm for thoughtful consideration of PM for non-psychiatric indications. This review seeks to be clinically relevant and is informed by the clinical experience of the authors. While this adds to the clinical applicability of the review, this introduces individual bias in the selection and interpretation of studies. However, given the broad scope and diversity of PM use across specialties, a narrative review allowed for assessment of clinically relevant examples without the constraints of a more systematic approach. As such, this review takes a pragmatic approach in acknowledging current trends in prescribing, safety aspects, and patient counseling considerations in both psychiatric and non-psychiatric specialties, even when high quality research evidence is lacking. Rather than limiting the review to only where the highest quality evidence was available, the authors made efforts to identify when evidence was limited and/or based primarily on low-quality evidence, while still providing insights into currently available best practices. However, this may have introduced bias in favor of studies supporting clinical benefit rather than negative studies, particularly as unpublished data/studies were not included.

## 6. Conclusions

Prescribing PMs can be particularly attractive to clinicians in cases involving complex somatic symptoms, treatment-resistant syndromes, or psychiatric–medical comorbidity, or for off-label indications based on knowledge and application of receptor pharmacology. However, even for primary psychiatric indications, response rates in placebo-controlled trials may be modest at best [66]. Moreover, the evidence base for off-label indications is often less robust than for FDA-approved indications, underscoring the need for ongoing, systematic evaluation of medication response, monitoring for side effects, and deprescribing when clear benefit is lacking. Responsible prescribing requires clinicians to seek out the most up to date literature and treatment guidelines when using PMs for off-label indications and must be aware of the full range of adverse effects and drug–drug interactions.

## Figures and Tables

**Table 1:** Principles of prescribing psychoactive medications.

1. Consider the range of dose options most appropriate for various indications (lower doses typically for pain or sleep, higher dosages for antidepressant effects)
2. Avoid therapeutic duplication (multiple antidepressants to treat different indications)
3. Avoid combining multiple serotonergic medications that may trigger serotonin syndrome
4. Be aware of “hidden” psychiatric medications and their potential side effects
5. Perform a thorough drug review to reduce risk of polypharmacy and drug–drug interactions
6. Provide detailed education to patients about both the psychiatric and non-psychiatric indications of the medications, so that patients are aware that prescribing an antidepressant does not necessarily mean they also have depression.

**Table 2:** Receptor profiles of common psychoactive medications.

Transporter or Receptor	Action *	Clinical Effects	Adverse Effects	Drug/Drug Class	Medical Uses
Serotonin reuptake inhibition	Increase serotonin	Antidepressant, Antianxiety,	Nausea, diarrhea, sexual dysfunction, serotonin syndrome (rare)	SSRIs, SNRIs, TCAs,	Premature ejaculation, IBS-C, limited usein migraine, refractory FM
Norepinephrine reuptake inhibition	Increase norepinephrine	Antidepressant, pain inhibition	Dry mouth, constipation, sweats, blood pressure elevation	SNRIs, TCAs, bupropion	Pain (migraine, neuropathic), Fibromyalgia, IBS-D
Dopamine reuptake inhibition	Increase dopamine	Antidepressant, CNS activation	Psychosis (rare), insomnia	Bupropion	Smoking cessation
5HT1A receptor (partial) agonism	Activity via the subtype of serotonin receptor	Antianxiety, improved gastric compliance	Nausea	Buspirone	Functional bloating and dyspepsia, shivering due to therapeutic normo-/hypothermia
5HT2A antagonism	Increased dopamine in striatum and pituitary	Antipsychotic, reduction in D2-mediated EPS)	Nausea, dizziness	Atypical antipsychotics, pimavanserin	Psychosis associated with Parkinson’s disease (pimavanserin)
5HT3 antagonism	Activity via the subtype of serotonin receptor	Antidepressant, antianxiety, reduced nausea and bloating	Weight gain, sedation	Mirtazapine, olanzapine, ondansetron	Chronic N/V syndrome, functional dyspepsia
Dopamine receptor antagonism	Decrease dopamine	Antipsychotic, antiemetic	EPS, weight gain	All antipsychotics, antiemetics (Compazine, promethazine, metoclopramide)	Nausea, hiccups
Monoamine oxidase inhibition	Increased serotonin, norepinephrine and dopamine	Antidepressant, antianxiety,	Hypertensive Crisis, hypotension, insomnia,	MAOI’s	Parkinson’s disease (MAO-B **)
Dopamine receptor agonist	Increase dopamine	Movement disorders	Nausea, sleepiness, impulse control behaviors, hallucinations	Ropinirole, pramipexole, bromocriptine	Parkinson’s disease, RLS, type II DM, NMS
Voltage-gated sodium channels	Inhibits the release of excitatory neurotransmitters	Anticonvulsant, antianxiety, analgesic	Dizziness, drowsiness, peripheral edema	Gabapentin, valproic acid	Pain, migraine, paroxysmal sympathetic hyperactivity
M1 antagonism	Blockade of muscarinic, type 1 receptor	Anticholinergic effects	Constipation, dry mouth, urinary retention, blurred visit	TCAs, paroxetine	Nocturnal enuresis, Parkinson’s disease, EPS
H1 antagonism	Antihistaminic	Sedation, antihistamine	Sedation, weight gain	TCAs, many antipsychotics, mirtazapine, diphenhydramine, hydroxyzine, trazodone	Insomnia
Alpha-1 adrenergic antagonism	Decreased adrenergic tone	Blood pressure lowered, decreased autonomic activity/arousal	Hypotension	TCAs, some antipsychotics, prazosin, trazodone	Various

* Action in part, as it relates to the medical uses, ** oral selegiline for treatment of Parkinson’s disease is selective for MAO-B when used at usual maximum dosing of 10 mg avoiding many of the side effects, interactions, and food restrictions of non-selective agents used for depression/anxiety.

## Data Availability

No new data were created or analyzed in this study.

## Abbreviations

GABA	Gamma-Aminobutyric Acid
CGRP	Calcitonin gene-related peptide
CNS	Central nervous system
ENS	Enteric nervous system
FDA	Food and Drug Administration
FGID	Functional gastrointestinal disorder
IBS	Irritable bowel syndrome
MAOI	Monoamine oxidase inhibitors
MG	Milligrams
PD	Parkinson’s disease
PMs	Psychotropic medications
SNRI	Serotonin and norepinephrine reuptake inhibitor
SSRI	Selective serotonin reuptake inhibitor
TCA	Tricyclic antidepressant

## References

[1] Colman D.R. (2006). The three princes of Serendip: Notes on a mysterious phenomenon. McGill J. Med..

[2] Ban T.A. (2007). Fifty years chlorpromazine: A historical perspective. Neuropsychiatr. Dis. Treat..

[3] Hillhouse T.M., Porter J.H. (2015). A brief history of the development of antidepressant drugs: From monoamines to glutamate. Exp. Clin. Psychopharmacol..

[4] Raskind M.A., Dobie D.J., Kanter E.D., Petrie E.C., Thompson C.E., Peskind E.R. (2000). The alpha1-adrenergic antagonist prazosin ameliorates combat trauma nightmares in veterans with posttraumatic stress disorder: A report of 4 cases. J. Clin. Psychiatry.

[5] Greenblatt D.J., Harmatz J.S., Shader R.I. (2018). Update on Psychotropic Drug Prescribing in the United States: 2014–2015. J. Clin. Psychopharmacol..

[6] Pratt L.A., Brody D.J., Gu Q. (2017). Antidepressant Use Among Persons Aged 12 and Over:United States, 2011–2014. NCHS Data Briefs.

[7] Richardson E. (2016). Off-Label Drug Promotion. Health Affairs Health Policy Brief.

[8] Strain J.J., Karim A., Caliendo G., Brodsky M., Lowe R.S., Himelein C. (2002). Neurologic drug-psychotropic drug update. Gen. Hosp. Psychiatry.

[9] Tajti J., Szok D., Csati A., Vecsei L. (2016). Prophylactic Drug Treatment of Migraine in Children and Adolescents: An Update. Curr. Pain Headache Rep..

[10] Sprenger T., Viana M., Tassorelli C. (2018). Current Prophylactic Medications for Migraine and Their Potential Mechanisms of Action. Neurotherapeutics.

[11] Jackson J.L., Cogbill E., Santana-Davila R., Eldredge C., Collier W., Gradall A., Sehgal N., Kuester J., Arias-Carrion O. (2015). A Comparative Effectiveness Meta-Analysis of Drugs for the Prophylaxis of Migraine Headache. PLoS ONE.

[12] Ozyalcin S.N., Talu G.K., Kiziltan E., Yucel B., Ertas M., Disci R. (2005). The efficacy and safety of venlafaxine in the prophylaxis of migraine. Headache.

[13] Xu X.M., Yang C., Liu Y., Dong M.X., Zou D.Z., Wei Y.D. (2017). Efficacy and feasibility of antidepressants for the prevention of migraine in adults: A meta-analysis. Eur. J. Neurol..

[14] Gomersall J.D., Stuart A. (1973). Amitriptyline in migraine prophylaxis. Changes in pattern of attacks during a controlled clinical trial. J. Neurol. Neurosurg. Psychiatry.

[15] Gallagher R.M., Mueller L.L., Freitag F.G. (2002). Divalproex sodium in the treatment of migraine and cluster headaches. J. Am. Osteopath. Assoc..

[16] Bussone G., Leone M., Peccarisi C., Micieli G., Granella F., Magri M., Manzoni G., Nappi G. (1990). Double blind comparison of lithium and verapamil in cluster headache prophylaxis. Headache.

[17] Honkaniemi J., Liimatainen S., Rainesalo S., Sulavuori S. (2006). Haloperidol in the acute treatment of migraine: A randomized, double-blind, placebo-controlled study. Headache.

[18] VanderPluym J.H., Halker Singh R.B., Urtecho M., Morrow A.S., Nayfeh T., Torres Roldan V.D., Farah M.H., Hasan B., Saadi S., Shah S. (2021). Acute Treatments for Episodic Migraine in Adults: A Systematic Review and Meta-analysis. JAMA.

[19] Peretz C., Segev H., Rozani V., Gurevich T., El-Ad B., Tsamir J., Giladi N. (2016). Comparison of Selegiline and Rasagiline Therapies in Parkinson Disease: A Real-life Study. Clin. Neuropharmacol..

[20] Rossano F., Caiazza C., Sobrino A., Solini N., Vellucci A., Zotti N., Fornaro M., Gillman K., Cattaneo C.I., Eynde V.V.D. (2023). Efficacy and safety of selegiline across different psychiatric disorders: A systematic review and meta-analysis of oral and transdermal formulations. Eur. Neuropsychopharmacol..

[21] Label: Rasagiline Mesulate Tablet. https://dailymed.nlm.nih.gov/dailymed/drugInfo.cfm?setid=7d070521-ea04-4483-bdb9-54a8859700df.

[22] Nayak L., Henchcliffe C. (2008). Rasagiline in treatment of Parkinson’s disease. Neuropsychiatr. Dis. Treat..

[23] Webster K.E., Harrington-Benton N.A., Judd O., Kaski D., Maarsingh O.R., MacKeith S., Ray J., Van Vugt V.A., Burton M.J. (2023). Pharmacological interventions for persistent postural-perceptual dizziness (PPPD). Cochrane Database Syst. Rev..

[24] Cha Y.H., Cui Y.Y., Baloh R.W. (2018). Comprehensive Clinical Profile of Mal De Debarquement Syndrome. Front. Neurol..

[25] Terry N., Margolis K.G. (2017). Serotonergic Mechanisms Regulating the GI Tract: Experimental Evidence and Therapeutic Relevance. Handb. Exp. Pharmacol..

[26] Mayer E.A. (2011). Gut feelings: The emerging biology of gut-brain communication. Nat. Rev. Neurosci..

[27] Lillestøl K. (2018). ‘Neurasthenia gastrica’ revisited: Perceptions of nerve-gut interactions in nervous exhaustion, 1880–1920. Microb. Ecol. Health Dis..

[28] Schmulson M.J., Drossman D.A. (2017). What Is New in Rome IV. J. Neurogastroenterol. Motil..

[29] Sobin W.H., Heinrich T.W., Drossman D.A. (2017). Central Neuromodulators for Treating Functional GI Disorders: A Primer. Am. J. Gastroenterol..

[30] Drossman D.A., Tack J., Ford A.C., Szigethy E., Tornblom H., Van Oudenhove L. (2018). Neuromodulators for Functional Gastrointestinal Disorders (Disorders of Gut-Brain Interaction): A Rome Foundation Working Team Report. Gastroenterology.

[31] Ford A.C., Lacy B.E., Harris L.A., Quigley E.M.M., Moayyedi P. (2019). Effect of Antidepressants and Psychological Therapies in Irritable Bowel Syndrome: An Updated Systematic Review and Meta-Analysis. Am. J. Gastroenterol..

[32] Cassell B., Gyawali C.P., Kushnir V.M., Gott B.M., Nix B.D., Sayuk G.S. (2015). Beliefs about GI medications and adherence to pharmacotherapy in functional GI disorder outpatients. Am. J. Gastroenterol..

[33] Glare P., Miller J., Nikolova T., Tickoo R. (2011). Treating nausea and vomiting in palliative care: A review. Clin. Interv. Aging.

[34] Fonte C., Fatigoni S., Roila F. (2015). A review of olanzapine as an antiemetic in chemotherapy-induced nausea and vomiting and in palliative care patients. Crit. Rev. Oncol. Hematol..

[35] Turk T., Liu C., Fujiwara E., Straube S., Hagtvedt R., Dennett L., Abba-Aji A., Dytoc M. (2023). Pharmacological Interventions for Primary Psychodermatologic Disorders: An Evidence Mapping and Appraisal of Randomized Controlled Trials. J. Cutan. Med. Surg..

[36] Gupta M.A., Gupta A.K. (2013). Cutaneous sensory disorder. Semin. Cutan. Med. Surg..

[37] Konstantinou G.N., Konstantinou G.N. (2019). Psychiatric comorbidity in chronic urticaria patients: A systematic review and meta-analysis. Clin. Transl. Allergy.

[38] Wong J.W., Koo J.Y. (2013). Psychopharmacological therapies in dermatology. Dermatol. Online J..

[39] Eskeland S., Halvorsen J.A., Tanum L. (2017). Antidepressants have Anti-inflammatory Effects that may be Relevant to Dermatology: A Systematic Review. Acta Derm. Venereol..

[40] Griffin J.R., Davis M.D. (2015). Amitriptyline/Ketamine as therapy for neuropathic pruritus and pain secondary to herpes zoster. J. Drugs Dermatol..

[41] McPhie M.L., Kirchhof M.G. (2022). A systematic review of antipsychotic agents for primary delusional infestation. J. Dermatol. Treat..

[42] Kouwenhoven T.A., van de Kerkhof P.C.M., Kamsteeg M. (2017). Use of oral antidepressants in patients with chronic pruritus: A systematic review. J. Am. Acad. Dermatol..

[43] Patel A., Jafferany M. (2020). Multidisciplinary and Holistic Models of Care for Patients With Dermatologic Disease and Psychosocial Comorbidity: A Systematic Review. JAMA Dermatol..

[44] Dahlhamer J., Lucas J., Zelaya C., Nahin R., Mackey S., DeBar L., Kerns R., Von Korff M., Porter L., Helmick C. (2018). Prevalence of Chronic Pain and High-Impact Chronic Pain Among Adults—United States, 2016. MMWR Morb. Mortal. Wkly. Rep..

[45] Max M.B., Culnane M., Schafer S.C., Gracely R.H., Walther D.J., Smoller B., Dubner R. (1987). Amitriptyline relieves diabetic neuropathy pain in patients with normal or depressed mood. Neurology.

[46] Fornasari D. (2017). Pharmacotherapy for Neuropathic Pain: A Review. Pain Ther..

[47] Sharp J., Keefe B. (2005). Psychiatry in chronic pain: A review and update. Curr. Psychiatry Rep..

[48] Hameroff S.R., Weiss J.L., Lerman J.C., Cork R.C., Watts K.S., Crago B.R., Neuman C.P., Womble J.R., Davis T.P. (1984). Doxepin’s effects on chronic pain and depression: A controlled study. J. Clin. Psychiatry.

[49] Patetsos E., Horjales-Araujo E. (2016). Treating Chronic Pain with SSRIs: What Do We Know?. Pain Res. Manag..

[50] Lynch M.E. (2001). Antidepressants as analgesics: A review of randomized controlled trials. J. Psychiatry Neurosci..

[51] Deng Y., Luo L., Hu Y., Fang K., Liu J. (2016). Clinical practice guidelines for the management of neuropathic pain: A systematic review. BMC Anesthesiol..

[52] Finnerup N.B., Attal N., Haroutounian S., McNicol E., Baron R., Dworkin R.H., Gilron I., Haanpää M., Hansson P., Jensen T.S. (2015). Pharmacotherapy for neuropathic pain in adults: A systematic review and meta-analysis. Lancet Neurol..

[53] Sansone R.A., Sansone L.A. (2014). Serotonin norepinephrine reuptake inhibitors: A pharmacological comparison. Innov. Clin. Neurosci..

[54] Fishbain D.A. (2005). Polypharmacy Treatment Approaches to the Psychiatric and Somatic Comorbidities Found in Patients with Chronic Pain. Am. J. Phys. Med. Rehabil..

[55] Schwan J., Sclafani J., Tawfik V.L. (2019). Chronic Pain Management in the Elderly. Anesthesiol. Clin..

[56] Uchida H. (2018). Neuroscience-based Nomenclature: What is it, why is it needed, and what comes next?. Psychiatry Clin. Neurosci..

[57] Ghinea N., Kerridge I., Little M., Lipworth W. (2017). Challenges to the validity of using medicine labels to categorize clinical behavior: An empirical and normative critique of “off-label” prescribing. J. Eval. Clin. Pract..

[58] Rusz C.M., Ősz B.E., Jîtcă G., Miklos A., Bătrînu M.G., Imre S. (2021). Off-Label Medication: From a Simple Concept to Complex Practical Aspects. Int. J. Environ. Res. Public Health.

[59] Gray S.L., Anderson M.L., Dublin S., Hanlon J.T., Hubbard R., Walker R., Yu O., Crane P.K., Larson E.B. (2015). Cumulative use of strong anticholinergics and incident dementia: A prospective cohort study. JAMA Intern. Med..

[60] Salahudeen M.S., Duffull S.B., Nishtala P.S. (2015). Anticholinergic burden quantified by anticholinergic risk scales and adverse outcomes in older people: A systematic review. BMC Geriatr..

[61] Molina K.C., Fairman K.A., Sclar D.A. (2018). Concomitant use of opioid medications with triptans or serotonergic antidepressants in US office-based physician visits. Drug Healthc. Patient Saf..

[62] Volpi-Abadie J., Kaye A.M., Kaye A.D. (2013). Serotonin syndrome. Ochsner J..

[63] Janowski J.P.B., Suarez L., Allen N.D., Sampson S.M. (2024). A Case Series of 11 Patients With Subacute Serotonin Syndrome. J. Acad. Consult. Liaison Psychiatry.

[64] Davidson K.W., Mangione C.M., Barry M.J., Nicholson W.K., Cabana M.D., Caughey A.B., Davis E.M., Donahue K.E., Doubeni C.A., US Preventive Services Task Force (2022). Collaboration and Shared Decision-Making Between Patients and Clinicians in Preventive Health Care Decisions and US Preventive Services Task Force Recommendations. JAMA.

[65] Raleigh M.F., Nelson M.D., Nguyen D.R. (2022). Shared Decision-Making: Guidelines From the National Institute for Health and Care Excellence. Am. Fam. Physician.

[66] Kirsch I. (2019). Placebo Effect in the Treatment of Depression and Anxiety. Front. Psychiatry.

